# Construction and validation of web-based nomograms for detecting and prognosticating in prostate adenocarcinoma with bone metastasis

**DOI:** 10.1038/s41598-022-23275-w

**Published:** 2022-11-03

**Authors:** Qiu Dong, Xiaoting Wu, Wenyi Gan, Tsz Ngai MOK, Juan Shen, Zhengang Zha, Junyuan Chen

**Affiliations:** 1 Center for Bone, Joint and Sports Medicine, The First Affiliated Hospital of Jinan University, Jinan University, Guangzhou, 510630 China; 2 Guangdong Provincial Key Laboratory of Pharmaceutical Bioactive Substances, Guangdong Pharmaceutical University, Guangzhou, 510006 China

**Keywords:** Cancer epidemiology, Cancer prevention, Cancer screening, Germ cell tumours, Metastasis, Prostate cancer, Epidemiology

## Abstract

Bone metastasis (BM) is one of the most common sites of metastasis in prostate adenocarcinoma (PA). PA with BM can significantly diminish patients’ quality of life and result in a poor prognosis. The objective of this study was to establish two web-based nomograms to estimate the risk and prognosis of BM in PA patients. From the Surveillance, Epidemiology, and End Results (SEER) database, data on 308,332 patients diagnosed with PA were retrieved retrospectively. Logistic and Cox regression, respectively, were used to determine independent risk and prognostic factors. Then, We constructed two web-based nomograms and the results were validated by receiver operating characteristic (ROC) curves, calibration curves, decision curve analysis (DCA) , and the Kaplan-Meier analyses. The independent risk factors for BM in PA patients included race, PSA, ISUP, T stage, N stage, brain, liver, lung metastasis, surgery, radiation and chemotherapy. The independent prognostic predictors for overall survival (OS) were age, marital status, PSA, ISUP and liver metastasis. Both nomograms could effectively predict risk and prognosis of BM in PA patients according to the results of ROC curves, calibration, and DCA in the training and validation sets. And the Kaplan-Meier analysis illustrated that the prognostic nomogram could significantly distinguish the population with different survival risks. We successfully constructed the two web-based nomograms for predicting the incidence of BM and the prognosis of PA patients with BM, which may assist clinicians in optimizing the establishment of individualized treatment programs and enhancing patients’ prognoses.

## Introduction

Prostate cancer is the most prevalent malignancy of the male reproductive system and the second leading cause of cancer-related mortality worldwide^1,2^. Adenocarcinoma is the most common pathological subtype of prostate cancer, accounting for 90% of cases, which usually develops in the glandular epithelium of the prostate’s periphery^1,3^. Due to the adoption of prostate specific antigen (PSA) testing for early detection of prostate adenocarcinoma (PA), significant improvements in PA survival rates have occurred, with a five-year survival rate of about 98% in the United States^4^. However, PSA testing remains not generally accessible globally, and as a consequence, many patients are diagnosed at advanced stage. More than 90% of advanced PA patients develop bone metastasis (BM), resulting in a significantly reduced median survival time of approximately 1.5–2 years^5–7^.

It was reported that 90% of patients who died from PA were associated with BM, representing a leading cause of morbidity and mortality among PA patients^8^. A majority of individuals with fatal PA have BM, and for most of these individuals, bone is the dominant or the only location of metastasis^9^. In addition, BM could cause a lot of skeletal-related events (SREs), such as pathological fractures, spinal cord compression, discomfort, hypercalcemia and the requirement for radiation treatment or surgery on the bone, which severely worsens the patient’s quality of life and prognosis^10,11^. Unfortunately, early-stage PA usually lacks evident symptoms, and some individuals seek care as a result of SERs. BM are most likely to occur in the pelvis, followed by the spine, while cranial metastases are rare. In the peripheral bones, metastases are most likely to occur in the extremities bones, with the femur being the most common. BM may induce systemic organ failure, which can lead to mortality in extreme instances, along with a number of SERs^12^. However, the precise mechanism of BM remains unknown as of today. It is believed to be a multi-step, multi-linked complex biological process in which multiple chemokines, integrins, adhesion factors, extracellular matrix, hematopoietic stem cells, and mesenchymal stem cells may be involved^13,14^. Consequently, it is vital to develop a reliable model for estimating the risk of BM in PA patients and the prognosis of PA patients with BM. Previous studies have found that age, marital status, PSA, TNM stage, International Society of Urological Pathology (ISUP) grade, surgery, radiation therapy, and chemotherapy are independent prognostic factors for PA patients^15–17^. To our knowledge, however, there are few studies exploring the relationship between clinicopathological features and the metastatic pattern of PA, and no prediction model has been constructed to predict the BM in PA or the prognosis of PA with BM.

Web-based nomogram has been widely used to assess the metastasis and prognosis of cancer patients due to its convenience and accuracy, making it an excellent option for our objective^18,19^. Therefore, we selected a representative cohort from the Surveillance, Epidemiology, and End Results (SEER) database to assess the incidence, risk factors, and prognosis of bone metastatic PA, as well as to develop two web-based nomograms for predicting BM in PA patients and overall survival (OS) of PA patients with BM, respectively.

## Patients and methods

The study protocol was examined by the First Affiliated Hospital of Jinan University Medical Ethics Committee and granted its exemption from ethical review. Meanwhile, formal informed consent was not required for this study, because all patients had already provided informed permission to be listed in the SEER database. This research was conducted and reported according to the TRIPOD declaration^20^.

### Patients

The PA patients for this study were obtained from the Surveillance, Epidemiology, and End Results (SEER) database between 2010 and 2015.The inclusion criteria were as follows: (1) patients were diagnosed according to the International Classification site for the prostate (C61.9) and ICD-O-3 histology codes for prostate carcinoma (8140)^21^; (2) patients were diagnosed between 2010 and 2015 based on their applicability to the 7th edition of the AJCC staging system; (3) the patient’s gender was confined to male only. Initially, we included 308,332 PA patients, of whom 13,647 (4.43%) had concurrent BM. The exclusion criteria were as follows: (1) patients were diagnosed only by autopsy or death certificate; (2) patients diagnosed without histological confirmation; (3) patients had more than one primary tumor; (4) patients’ follow-up information were incomplete; (5) patients’ baseline demographic data were unknown or incomplete; (6) patients’ clinicopathological parameter data were unknown or incomplete. Finally, 132,601 individuals diagnosed with PA were included in the present research, including 4147 with BM. All patients were used to form a diagnostic cohort to explore the risk factors of BM and construct a predictive nomogram. Subsequently, 4147 PA patients with BM were employed to establish a prognostic cohort in order to explore the prognostic characteristics for BM patients and build a novel prognostic nomogram.

**Figure 1:**
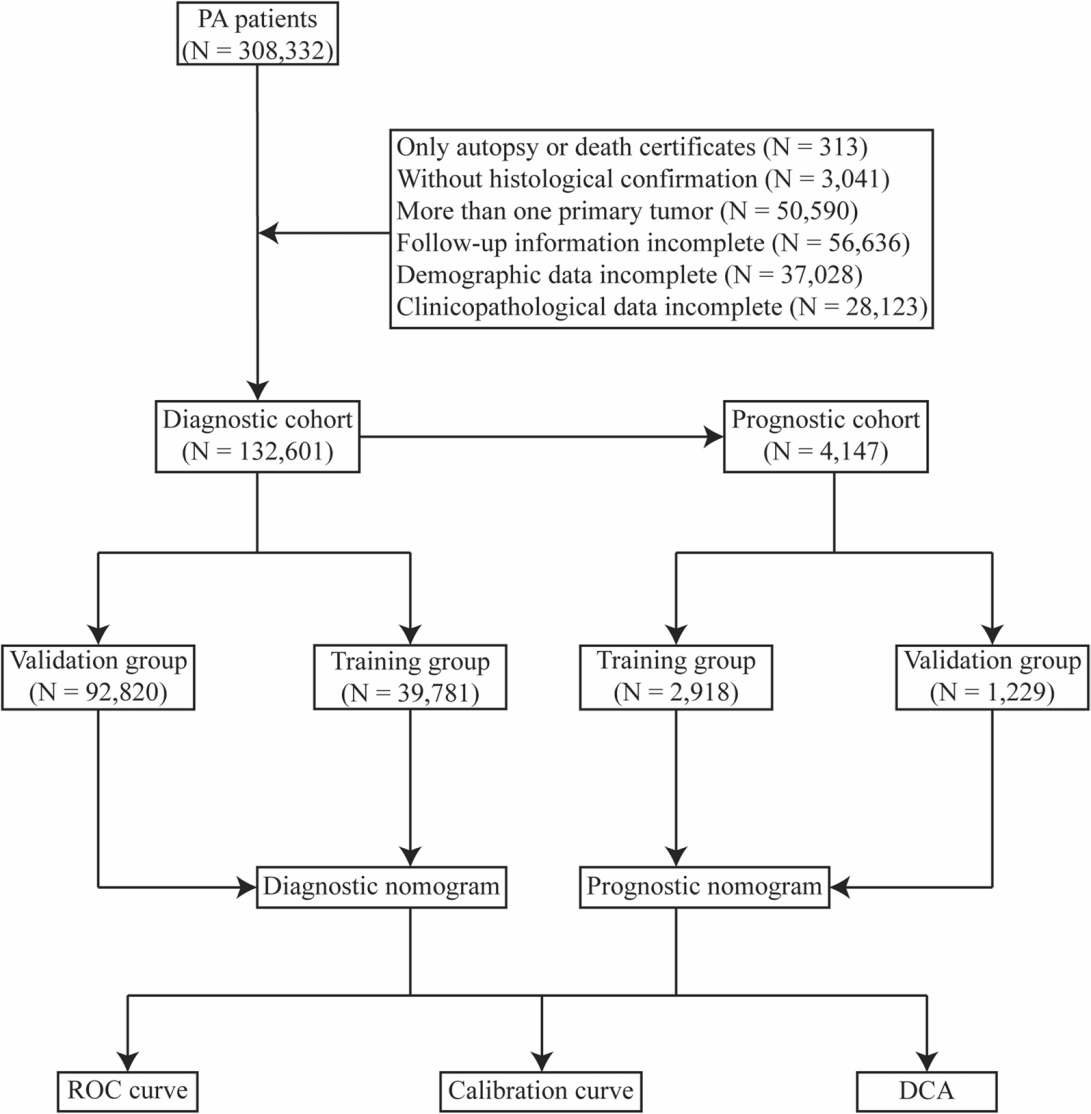
Flowchart of patient recruitment.

In the diagnostic cohort, 70% of the subjects were randomly assigned to the training group, and 30% of the subjects were assigned to the validation group. While the training and validation groups for the prognostic cohort were comprised of PA patients with BM from the respective diagnostic cohort groups. For each cohort, the training group was employed to construct the nomogram, while the validation group was utilized to validate it. The specific screening process was shown in Fig. 1.

### Data collection

In this study, the following characteristics were used to determine risk factors for BM in PA patients: age, race, marital status, grade, PSA, ISUP,T stage, N stage, brain, liver and lung metastasis. In addition, we identified the prognostic characteristics of PA patients with BM by assessment of their survivability.

Our research also conducted survival analyses to investigate prognostic factors of PA patients with BM. Based on the mentioned variables, three treatment factors were taken into consideration: surgery, radiotherapy, and chemotherapy. In this section, the primary outcome was OS, which was defined as the period between the data of diagnosis and the data of death for any reason.

### Statistical analysis

In the present study, we followed the methods of chen et al. 2021^22^. All statistical analysis was conducted using R software (version 4.2.1), and a two-sided P value <0.05 was regarded as statistically significant. All PA patients were randomly divided into training and validation groups. Then the Chi-square test or Fisher’s exact test was performed to compare the distribution of variables between the training and validation groups.

Firstly, the X-tile software was used to determine the optimal age cut-off values, which were divided into young (<75 years) and elderly ($\ge$75 years) groups (Supplementary Fig. S1). In the diagnostic cohort, multicollinearity was displayed as a heat map of correlation variables related to BM using the ’corrplot’ package. To prevent overfitting, the ’glmnet’ package was used for the least absolute shrinkage and selection operator (LASSO) regression model to determine the essential variables of BM. Using the representative variables chosen by LASSO, a multivariate stepwise logistic regression model was then established to identify the independent risk factors for BM. Meanwhile, the forest plots denoting odd ratio (OR) and 95% confidence intervals (CI) were plotted by R package ‘forestplot’. In addition, the ’regplot’ package wes utilized to build a novel diagnostic nomogram. Subsequently, we developed the dynamic nomogram using the package ’DynNom’.The receiver operating characteristic (ROC) curve of the nomogram and all independent variables was constructed, and the area under the curve (AUC) was evaluated to assess the discrimination using ’pROC’ package. Furthermore, calibration curves and decision curve analysis (DCA) were used to assess the accuracy of the nomogram using ’rms’ and ’ggDCA’ packages, respectively.

In the prognostic cohort, similar heat maps were generated to assess the multicollinearity of the relevant factors. For the construction of multivariate Cox regression analysis, we selected a model within one standard error of the best-performing lambda (lambda.1se) based on LASSO regression, by ’glmnet’ and ’survival’ packages. Then, we built forest plots representing hazard ratio (HR) and 95% CI. Both nomograms and web-based nomograms were created to forecast the OS of PA patients with BM, while nomograms were applied to compute individual risk scores. Furthermore, time-dependent ROC curves were plotted for the nomograms and all independent prognostic factors at 1, 3 and 5 years were computed, and the associated time-dependent AUC values were employed to demonstrate discriminatory power using ’survivalROC’ package. To analyze the nomograms, calibration curves and DCA were presented at 1, 3, and 5 years. Individuals were split into low or high risk groups using the X-tile software based on the values of the risk score (Supplementary Fig. S2). The difference in OS status between the two groups was demonstrated using Kaplan–Meier (KM) survival curves with a log-rank test by ’survminer’ package.

## Results

### Baseline characteristics of the study cohort

In total, 132,601 PA patients were included in this study and randomly assigned into training (n = 92,820) and validation groups (n = 39,781). The majority of PA patients were young (89.7%), white (75.1%) and married (75.3%). The grade of PA was predominantly grade II (41.0%) and grade III (48.6%). The majority of patients had PSA level <10 ng/mL (74.0%) and were categorized as ISUP grade 1 (40.1%). The most common T and N stage were T1 (43.0%), T2 (43.7%), and N0 (96.7%), respectively. Regarding distant metastasis, most PA patients without BM (96.9%), brain metastasis (100.0%), liver metastasis (99.9%), and lung metastasis (99.8%). Meanwhile, the p-value indicates that the deviation between the two groups was randomly distributed (Table 1).

**Table 1:** Baseline clinical characteristics of PA patients.

	Training (N = 92,820)	Validation (N = 39,781)	Overall (N = 132,601)	X$^2$	P
**Age**				1.99	0.16
Young	83,374 (89.8%)	35,630 (89.6%)	119,004 (89.7%)		
Elderly	9446 (10.2%)	4151 (10.4%)	13,597 (10.3%)		
**Race**				0.07	0.96
White	69,668 (75.1%)	29,849 (75.0%)	99,517 (75.1%)		
Black	18,176 (19.6%)	7785 (19.6%)	25,961 (19.6%)		
Other	4976 (5.4%)	2147 (5.4%)	7123 (5.4%)		
**Marital status**				5.17	0.02
Married	70,037 (75.5%)	29,782 (74.9%)	99,819 (75.3%)		
Unmarried	22,783 (24.5%)	9999 (25.1%)	32,782 (24.7%)		
**Grade**					
I	9517 (10.3%)	4173 (10.5%)	13,690 (10.3%)		
II	38,005 (40.9%)	16,299 (41.0%)	54,304 (41.0%)		
III	45,187 (48.7%)	19,269 (48.4%)	64,456 (48.6%)		
IV	111 (0.1%)	40 (0.1%)	151 (0.1%)		
**PSA (ng/ml)**				2.52	0.28
$<10$	68,621 (73.9%)	29,546 (74.3%)	98,167 (74.0%)		
10–20	14,525 (15.6%)	6197 (15.6%)	20,722 (15.6%)		
$>20$	9674 (10.4%)	4038 (10.2%)	13,712 (10.3%)		
**ISUP group**				5.36	0.25
1	37,162 (40.0%)	16,036 (40.3%)	53,198 (40.1%)		
2	26,079 (28.1%)	11,231 (28.2%)	37,310 (28.1%)		
3	12,073 (13.0%)	5081 (12.8%)	17,154 (12.9%)		
4	9513 (10.2%)	3950 (9.9%)	13,463 (10.2%)		
5	7993 (8.6%)	3483 (8.8%)	11,476 (8.7%)		
**T stage**				4.84	0.18
T1	39,719 (42.8%)	17,244 (43.3%)	56,963 (43.0%)		
T2	40,597 (43.7%)	17,285 (43.5%)	57,882 (43.7%)		
T3	11,707 (12.6%)	4898 (12.3%)	16,605 (12.5%)		
T4	797 (0.9%)	354 (0.9%)	1151 (0.9%)		
**N stage**				0.24	0.62
N0	89,701 (96.6%)	38,466 (96.7%)	128,167 (96.7%)		
N1	3119 (3.4%)	1315 (3.3%)	4434 (3.3%)		
**Bone metastasis**				0.25	0.61
No	89,902 (96.9%)	38,552 (96.9%)	128,454 (96.9%)		
Yes	2918 (3.1%)	1229 (3.1%)	4147 (3.1%)		
**Brain metastasis**				0.54	0.46
No	92,800 (100.0%)	39,769 (100.0%)	132,569 (100.0%)		
Yes	20 (0.0%)	12 (0.0%)	32 (0.0%)		
**Liver metastasis**				0.01	0.92
No	92,709 (99.9%)	39,735 (99.9%)	132,444 (99.9%)		
Yes	111 (0.1%)	46 (0.1%)	157 (0.1%)		
**Lung metastasis**				0.34	0.56
No	92,620 (99.8%)	39,688 (99.8%)	132,308 (99.8%)		
Yes	200 (0.2%)	93 (0.2%)	293 (0.2%)		

In the prognostic cohort, 4147 eligible PA patients with BM were used to explore related variables. Compared with individuals without BM, the majority of clinicopathological features were grade III (91.2%), PSA level >20 ng/mL (76.9%), and ISUP 5 (58.6%). The vast majority of patients did not undergo surgery (86.1%), and neither radiation (75.0%) nor chemotherapy (87.3%) was administered. Likewise, there was no statistically significant difference between the training and validation sets (Table 2).

**Table 2:** Baseline clinical characteristics of PA patients with BM.

	Training (N = 2918)	Validation (N = 1229)	Overall (N = 4147)	X$^2$	P
**Age**				0.86	0.35
Young	2135 (73.2%)	917 (74.6%)	3052 (73.6%)		
Elderly	783 (26.8%)	312 (25.4%)	1095 (26.4%)		
**Race**				2.38	0.3
White	2072 (71.0%)	885 (72.0%)	2957 (71.3%)		
Black	647 (22.2%)	276 (22.5%)	923 (22.3%)		
Other	199 (6.8%)	68 (5.5%)	267 (6.4%)		
**Marital status**				0.67	0.41
Married	1817 (62.3%)	748 (60.9%)	2565 (61.9%)		
Unmarried	1101 (37.7%)	481 (39.1%)	1582 (38.1%)		
**Grade**				4.66	0.2
I	18 (0.6%)	11 (0.9%)	29 (0.7%)		
II	223 (7.6%)	84 (6.8%)	307 (7.4%)		
III	2654 (91.0%)	1130 (91.9%)	3784 (91.2%)		
IV	23 (0.8%)	4 (0.3%)	27 (0.7%)		
**PSA (ng/ml)**				0.47	0.79
$<10$	327 (11.2%)	138 (11.2%)	465 (11.2%)		
10–20	352 (12.1%)	139 (11.3%)	491 (11.8%)		
$>20$	2239 (76.7%)	952 (77.5%)	3191 (76.9%)		
**ISUP**				10.02	0.04
1	58 (2.0%)	35 (2.8%)	93 (2.2%)		
2	160 (5.5%)	78 (6.3%)	238 (5.7%)		
3	284 (9.7%)	89 (7.2%)	373 (9.0%)		
4	710 (24.3%)	304 (24.7%)	1014 (24.5%)		
5	1706 (58.5%)	723 (58.8%)	2429 (58.6%)		
**T stage**				1.4	0.71
T1	1103 (37.8%)	460 (37.4%)	1563 (37.7%)		
T2	1003 (34.4%)	444 (36.1%)	1447 (34.9%)		
T3	436 (14.9%)	175 (14.2%)	611 (14.7%)		
T4	376 (12.9%)	150 (12.2%)	526 (12.7%)		
**N stage**				0.57	0.45
N0	2008 (68.8%)	861 (70.1%)	2869 (69.2%)		
N1	910 (31.2%)	368 (29.9%)	1278 (30.8%)		
**Brain metastasis**				<0.01	1
No	2903 (99.5%)	1221 (99.3%)	4124 (99.4%)		
Yes	15 (0.5%)	8 (0.7%)	23 (0.6%)		
**Liver metastasis**				0.01	0.75
No	2831 (97.0%)	1193 (97.1%)	4024 (97.0%)		
Yes	87 (3.0%)	36 (2.9%)	123 (3.0%)		
**Lung metastasis**				0.84	0.36
No	2758 (94.5%)	1152 (93.7%)	3910 (94.3%)		
Yes	160 (5.5%)	77 (6.3%)	237 (5.7%)		
**Surgery**				0.93	0.34
No	2523 (86.5%)	1048 (85.3%)	3571 (86.1%)		
Yes	395 (13.5%)	181 (14.7%)	576 (13.9%)		
**Radiation**				1.3	0.25
No/Unknown	2174 (74.5%)	937 (76.2%)	3111 (75.0%)		
Yes	744 (25.5%)	292 (23.8%)	1036 (25.0%)		
**Chemotherapy**					
No/Unknown	2549 (87.4%)	1073 (87.3%)	3,622 (87.3%)	<0.01	1
Yes	369 (12.6%)	156 (12.7%)	525 (12.7%)		

### Correlations among characteristics

**Figure 2:**
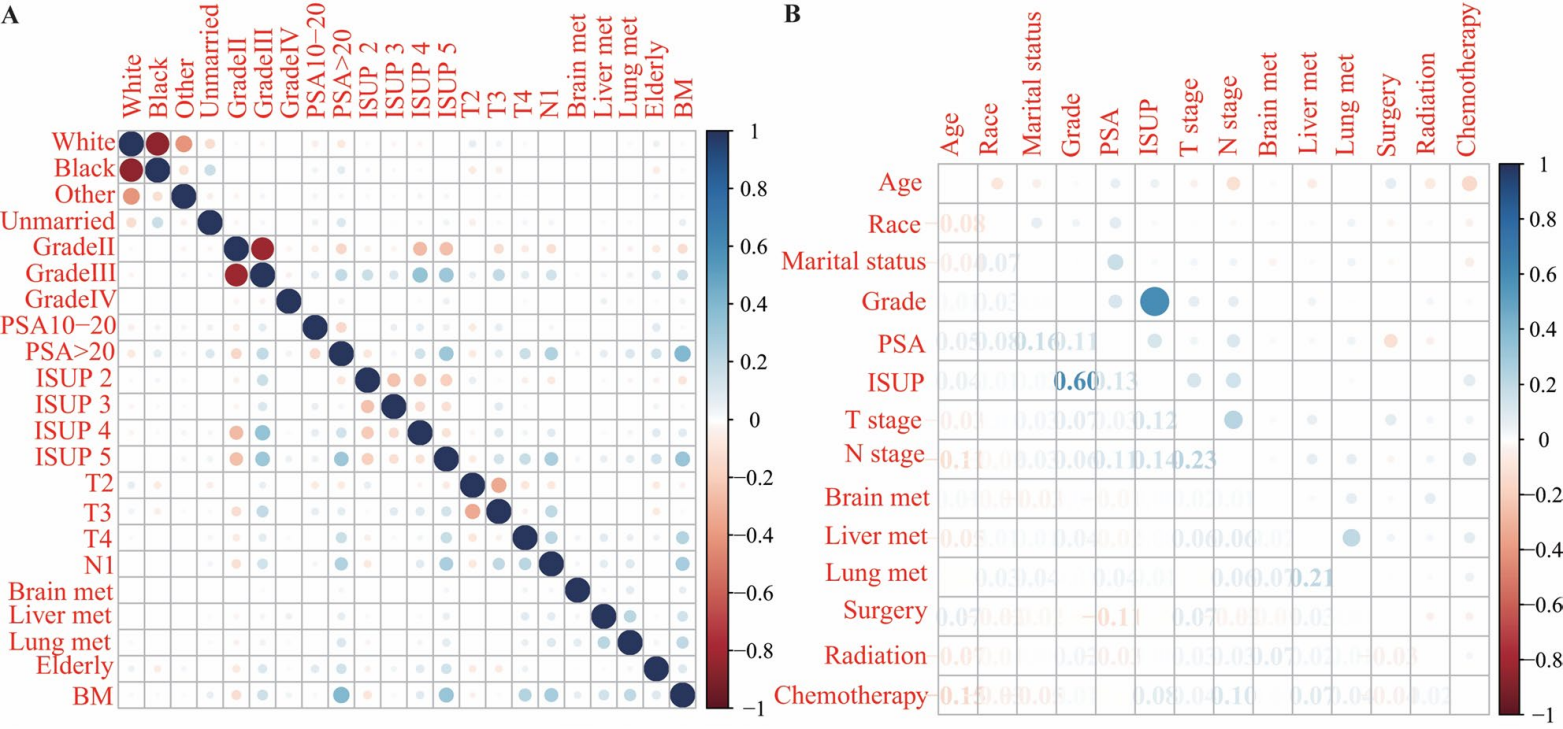
The heat maps of correlation analysis results between all included characteristics. **(A)** The results of the correlation analysis of the characteristics included in the diagnostic cohort. **(B)** The results of the correlation analysis of the characteristics included in the prognostic cohort.

In the diagnostic cohort, before establishing logistic regression, Spearman’s correlation was utilized to guarantee that no collinearity occurred between screened characteristics (Fig. 2A). While the multicollinearity of the variables was investigated in the prognostic cohort, no significant correlations (correlation coefficient $\ge$0.70) were reported (Fig. 2B)^23^.

### Diagnostic nomogram development and validation

**Figure 3:**
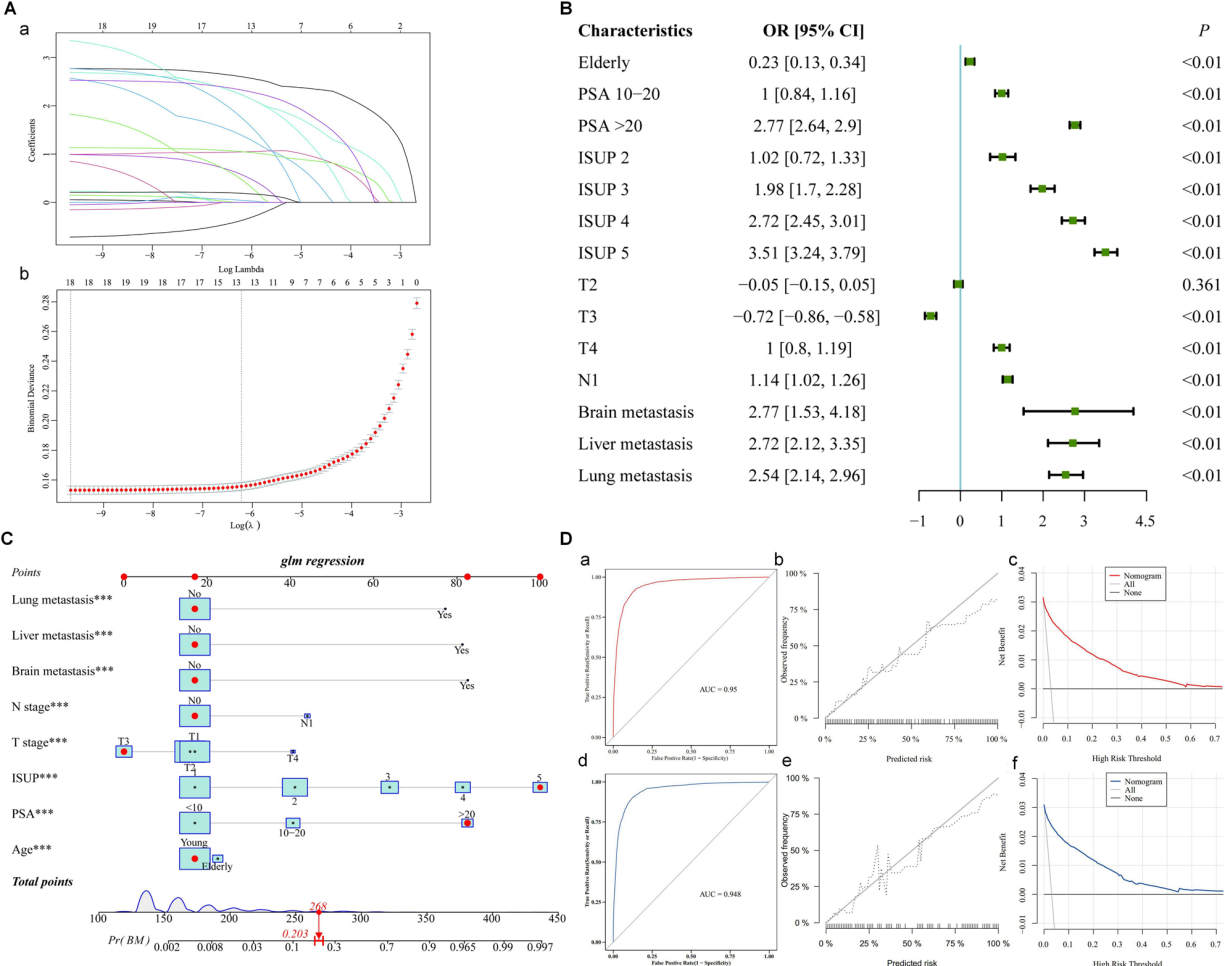
**(A)** Clinical features selection by the LASSO binary logistic regression model. **(B)** Forest plot of data from multivariate logistic regression revealing factors independently associated with the BM of PA patients. **(C)** The diagnostic nomogram to estimate the risk of BM in PA patients. **(D)** Validation of the diagnostic nomogram. The ROC **(a)**, calibration curve **(b)**, and DCA **(c)** of the training group, and the ROC **(d)**, calibration curve **(e)**, and DCA **(f)** of the validation group.

In the diagnostic cohort, there were 132,601 PA patients, and 4147 (3.1%) of them confirmed as BM. LASSO regression analysis was used to select the predictor clinical features from Table 1, and multivariate logistic regression was used to establish the prediction model. The prediction model contained eight of the original 12 variables, namely, age, PSA, ISUP, T stage, N stage, brain metastasis, liver metastasis, and lung metastasis (Fig. 3). These eight variables exhibited nonzero coefficients in the LASSO regression, which could be used to objectively predict the probability of BM among PA patients. In addition, the multivariate logistic regression analysis determined that elderly, higher PSA level, higher ISUP grade, higher T stage, higher N stage, brain metastasis, liver metastasis, and lung metastasis were independent risk predictors of BM in primary PA patients (Fig. 3B).

On the basis of these eight independent variables, we developed a novel nomogram and a web-based tool for predicting the risk of BM in PA patients (Fig. 3C) (https://dongnomogram.shinyapps.io/BM_Risk_Nomogram/). Then, the ROC curves for the training and validation sets were established, and the AUC of the nomogram for the training and validation sets was 0.95 and 0.948, respectively (Fig. 3Da,d). AUC of 1.0 denoted an ideal classifier, whereas AUC $\ge$0.5 denoted a classifier that outperformed random^24^. All AUC values of diagnostic nomogram were greater than 0.9, indicating the high performance of our model in predicting BM. Meanwhile, the calibration curves of the nomogram demonstrated high consistency between the observed and predicted results (Fig. 3Db,e). In addition, the DCA curves revealed positive net benefits, demonstrating the improved diagnostic precision of the nomogram (Fig. 3Dc,f).

### Prognostic nomogram development and validation

**Figure 4:**
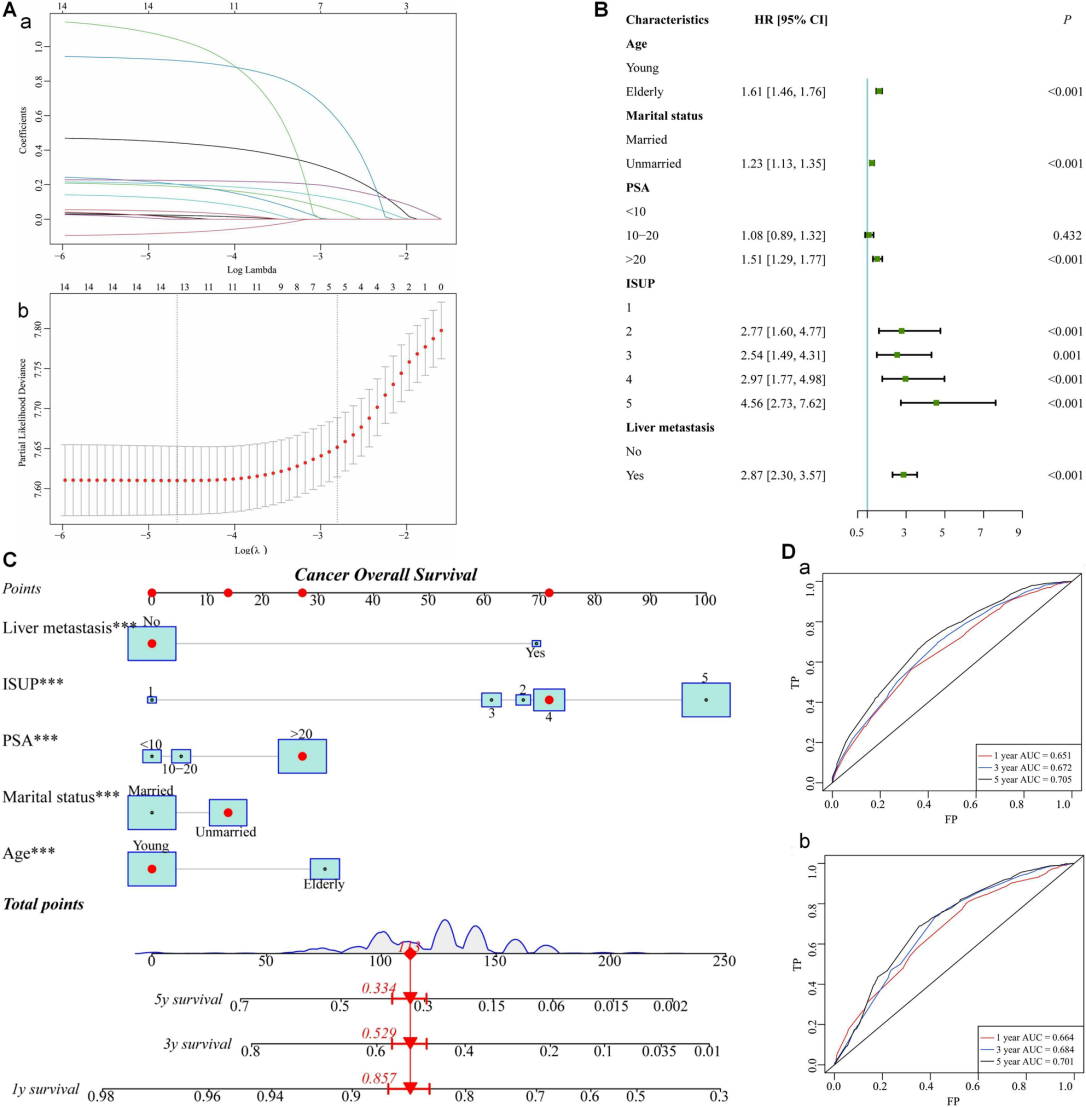
**(A)** LASSO Cox regression model construction. **(B)** Forest plot of data of independent risk factors in multivariate Cox regression. **(C)** The prognostic nomogram to estimate the risk of BM in PA patients. **(D)** Time-dependent ROC curves analysis of the prognostic nomogram for the 1, 3, and 5 years in the training set **(a)** and the validation set **(b)**.

In the prognostic cohort, the LASSO regression analysis identified five predictive variables from those shown in Table 2 that might be included in the multivariate Cox analysis (Fig. 4A). At the same time, the test of proportional hazards assumption indicated that the proportional hazards assumption was not violated (Supplementary Fig. S3). According to the results of multivariate Cox regression analysis, age, marital status, PSA level, ISUP group, and liver metastasis were independent prognostic variables (Fig. 4B). Based on the five prognostic factors, we constructed a novel nomogram to predict the OS of PA patients with BM (Fig. 4C). Additionally, we developed a straightforward web-based tool for predicting the prognosis of PA patients with BM (https://dongnomogram.shinyapps.io/Cancer_Overall_Survival/).

**Figure 5:**
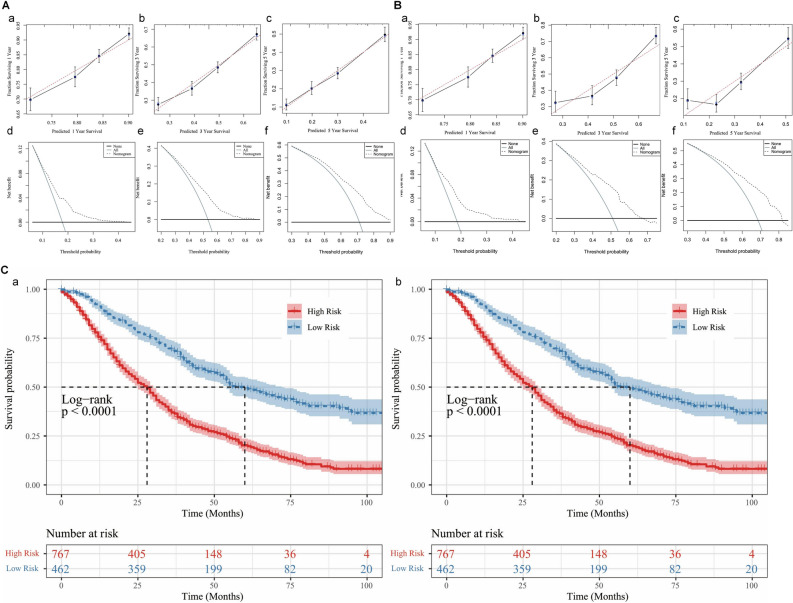
**(A)** The calibration curves of the nomogram for the 1 **(a)**, 3 **(b)**, and 5 years **(c)** in the training set. The decision curve analysis of the nomogram for the 1 **(d)**, 3 **(e)**, and 5 years **(f)** in the training set. **(B)** The calibration curves of the nomogram for the 1 **(a)**, 3 **(b)**, and 5 years **(c)** in the validation set. The decision curve analysis of the nomogram for the 1 **(d)**, 3 **(e)**, and 5 years **(f)** in the validation set. **(C)** The Kaplan–Meier survival curves of OS for low– and high–risk patients in the training set **(a)**, pediatric validation set **(b)**.

Time-dependent ROC curves indicated that the AUC values at 1, 3, and 5 years were 0.651, 0.672, and 0.705 in the training set (Fig. 4Da), and 0.664, 0.684, and 0.701 in the validation set (Fig. 4Db). All the AUC values were greater than 0.5, demonstrating the nomogram has some predictive performance. Furthermore, the AUC values increased with time, demonstrating the accuracy of our model in predicting the prognosis of advanced PA. Meanwhile the calibration curves of the nomogram for the 1, 3, and 5 years OS showed an excellent level of concordance between the predicted probability and the actual observation in the training group (Fig. 5Aa–c) and validation group (Fig. 5Ba–c). Meanwhile, the DCA curves proved that the nomogram had high potential for clinical utility (Fig. 5Ad–f,  Bd–f). According to the optimal cut-off value of the risk score, the low risk group consisted of scores between 0 and 113, while the high risk group consisted of values between 114 and 241 (Supplementary Fig. S2). Moreover, KM survival curves showed that the OS of patients in the low risk group was superior to that of patients in the high risk group (Fig. 5C).

## Discussion

Advanced PA is dominant by BM, which usually be osteogenic lesions, causing structurally disturbed and unstable osteogenic changes^25^. The pathogenesis of prostate cancer bone metastases is now thought to include two major mechanisms: metastasis to the spine via the Baston spinal venous plexus and Paget’s seed and soil theory; nevertheless, the specific mode of action is still being investigated. In 2020, PA have affected roughly 1.41 million men globally, accounting for 30.7% of all cancers diagnosed^1^. The most prevalent location of PA distant metastasis is the bone. It was reported that the incidence of BM was about 3%–10% in patients who were initially diagnosed with PA in developed countries, while it could reach 27% in developing countries^26,27^. This study found that the probability of BM in patients with PA was 3.1% (4147/132,601), which is comparable with results from previous research. Since early PA symptoms are similar to those of benign prostatic hyperplasia, many individuals have BM when they are first diagnosed. Patients with only BM have a better prognosis than those with multiple sites. However, until patients have BM, their OS will decrease rapidly, with 1- and 5-year survival rates of 47% and 3%, respectively^12^. BM patients are often untreatable with standard treatments (surgery, radiation, and chemotherapy) and may experience a succession of skeletal-related events which diminish their quality of life^23^. Therefore, we must discover the effective risk and prognostic factors for BM in patients with PA for early diagnosis, to facilitate early prevention, and to assess the prognosis of BM in patients with PA. In this study, we constructed a diagnostic nomogram to predict BM in newly diagnosed PA patients and a prognostic nomogram for BM patients. By obtaining data on several key accessible variables on the nomogram, diagnosis-related and prognosis-related scores can be calculated, thus facilitating further clinical assessment and management.

Based on the seed and soil theory, PA disseminates to bone via the hematogenous route, and the microenvironment of bone provides a particularly fertile environment for tumor cell proliferation and progression^28^. PA cells have a subtle tendency to bone, and in an autopsy study, 90.1% of individuals who died of PA were diagnosed with metastatic cancer to bone^29^. However, PA metastasis to the bone is a complicated progression, and its exact mechanisms remain unknown. It has been shown that bone-derived chemokines operate as chemoattractants for circulating PA cells, which, upon arriving in bone, are exposed to elements within the bone microenvironment that promote the establishment of metastasis. The release of growth factors by tumor cells may directly promote osteoblast activity, leading to a rise in receptor activator of NF-$\kappa$B (RANK) ligands expression. This overproduction of RANK ligand then mediates a vicious cycle of tumor growth and bone destruction by promoting the formation, function, and survival of osteoclasts, which results in excessive bone reabsorption, and the release of growth factors from the bone matrix, which may perpetuate tumor activity^30,31^. Additionally, as for clinical characteristics, research discovered that BM in PA patients had a substantial correlation with PSA^32^, T-stage^33^, and ISUP groups^34^. When PSA <10ng/ml, the frequency of bone metastases in PA patients was found to be close to zero; when PSA >20ng/ml, the probability of bone metastases was reported to be over 70%^32^. According to the European Urological, the risk of newly diagnosed PA patients were stratified into low risk (Gleason score $\le$7, T1–T3, and PSA <10ng/ml, with T1 patients considered low risk regardless of PSA value), intermediate risk (Gleason score $\le$7, T2/T3, and PSA >10ng/mL), or high risk (Gleason >7 score)^35^. The Gleason score has been utilized for half a century to estimate the prognosis of PA patients and to guide treatment decisions. The grading system has been the subject of extensive research, which has influenced its use in clinical practice. Subsequently, ISUP convened several consensus to modify both the grading standards and the manner in which grades were presented in accordance with the Gleason score^36,37^. Ultimately, to more effectively communicate the prognostic significance of PA, the ISUP Consensus Conference established a five-grade grading system, with grades 1 to 5 based on Gleason scores $\le$6, $3+4=7, 4+3=7, 8$, and 9–10, respectively. In this study, we used clinical data from the SEER database for analysis to identify eight predictors of BM in PA patients, namely, age, PSA, T classification, N grade, brain metastases, liver metastases, and lung metastases. The association between PSA value, T grade and ISUP grade and BM in PA patients has been confirmed in previous research. Surprisingly, however, T3 stage PA patients had the least risk of BM. We hypothesize that it may be connected to the PSA value; when PSA <10 ng/ml, patients with stage T1–T3 belong to the low risk group in total. Meanwhile, our research revealed that older individuals, N1 stage, brain metastases, liver metastases, and lung metastases were more likely to develop BM. We speculate that this is owing to the weakened immune of elderly patients, which makes them susceptible to BM. Moreover, we found lymph node, brain metastasis, liver metastasis and lung metastasis are risk factors for synchronous BM.

Currently, there is no curative therapy for PA patients with BM, and SERs are highly prevalent; therefore, early detection of BM is crucial for patients to receive appropriate treatment to reduce the inconvenience and pain caused by various complications, allowing them to live with tumors for an extended period of time. To date, the majority of research focused only on independent risk variables, and only one realistic model has been developed to predict the risk of BM in PA patients^38^. In the prior model, practitioners were required to precisely estimate the prostate’s volume to forecast BM, which was impractical for treatment. To answer this deficiency, we developed a novel web-based nomogram based on eight independent predictors and demonstrated excellent performance with ROC curves, calibration curves, and DCA, which may improve the current state of risk assessment and enable more accurate personalized clinical decision making.

Most PA patients with BM didn’t exhibit overt clinical symptoms in the early stages, and some individuals might not be identified until they present with impaired limb movement, bone pain and pathologic fractures^39^. The spine is the most typical location for BM, which may induce spinal discomfort, radiating pain, limb paralysis, and even paraplegia in extreme cases. Patients with extensive BM may also have systemic symptoms, including weariness, wasting, anemia, and possibly multiorgan system failure. In addition, hypercalcemia may affect numerous physiological systems, such as the neurological system, cardiovascular system, digestive system, urinary system, and even tumor cachexia. Although SREs are commonly used to describe the specific symptoms of BM in current clinical practice, the concept of SREs originated in early clinical studies of bone-modifying drugs and was only used as a clinical endpoint to assess the efficacy of drug therapy, including four types of pathological fractures, spinal cord compression, bone surgery, and bone radiotherapy^40^. Although there is a correlation between SREs and clinical symptoms, the subjective evaluation procedure and the fact that it may be altered in the short term make it inappropriate as an endpoint in clinical trials. Therefore, OS was chosen as the outcome measure for BM patients. At present, the primary objectives of treating PA patients with BM are to prevent and minimize the incidence of SREs, alleviate the pain caused by BM, and enhance the quality of life of the patients. Our research found that OS in PA patients was associated with five factors: age, marital status, PSA value, ISUP group, and liver metastases, rather than treatment methods such surgery, chemotherapy, or radiation therapy. Furthermore, using ROC curves, calibration curves, and DCA validation, it was shown that the nogram may provide new opportunities for individualized evaluation and clinical decision-making. This conclusion is comparable to the metastatic PA model developed by Jiang et al.^41^. It seems that younger, married, lower PSA levels, lower ISUP grades, and without liver metastases individuals achieved better OS. However, grade 3 of the ISUP had a higher OS than grade 2, which is unexpected. This may be because both have comparable Gleason scores and largely intact prostate tissue. Nonetheless, ISUP grade 3 had a higher OS than grade 2, which may be due to the fact that both grades have comparable Gleason scores and partly intact prostate tissue. In addition, Hu et al.^42^ established the prognostic nomogram of PA patients with BM using six genes signatures, which is more costly and cumbersome than our model.

Our study has a significant advantage compared with previous similar studies. First, the subject of our study is not consisted with previous research. The majority of previous research focused on the risk and prognosis of PA patients with BM^38,39,41^. However since PA is a heterogeneous disease with various biological traits for different pathological subtypes, we selected PA as our study topic. Second, our study had a considerable sample size, and to the best of our knowledge, it was the largest sample size focusing on PA patients with BM. Third, we developed two practical web-based tools to aid clinicians in their daily work by allowing for more efficient and easy prediction of BM risk and prognosis in PA patients.

## Limitations

Nevertheless, we should acknowledge that there are some limitations in our work. Firstly, although we built the models using the training sets and verified them with the validation sets, all data came from the SEER database, which might introduce inherent bias. Second, since the information in the SEER database was about the disease at the time of the first diagnosis, it cannot cover BM that occurred later in the course of disease. Finally, we only had information on surgery, radiation, and chemotherapy and lacked particular information on systemic treatment or even SREs, thus more research was required for follow-up.

## Conclusion

Our findings showed that age, PSA level, ISUP group, T stage, brain metastasis, liver metastasis, and lung metastasis were independent risk factors of BM for PA patients. And age, marital status, PSA level, ISUP group, and liver metastasis were revealed to be the independent prognostic factors for PA patients with BM. Two nomograms may be utilized as accessible graphical tools to objectively assess the risk and prognosis of PA patients with BM, which could improve clinical benefit as well cost-effectiveness in the management. And, these two web-based nomograms may assist clinicians in optimizing the establishment of individualized treatment programs and boosting patients’ prognosis.

## Supplementary Information

Supplementary Figure S1.

Supplementary Figure S2.

Supplementary Figure S3.

## Data Availability

The datasets generated and/or analyzed for this study can be available in the SEER dataset repository (https://seer.cancer.gov/).

## Abbreviations

AUC: Area under the curve
BM: Bone metastasis
CI: Confidence intervals
DCA: Decision curve analysis
HR: Hazard ratio
ISUP: International Society of Urological Pathology
KM: Kaplan–Meier
LASSO: Least absolute shrinkage and selection operator
OR: Odd ratio
OS: Overall survival
PA: Prostate adenocarcinoma
PSA: Prostate specific antigen
ROC: Receiver operating characteristic
SEER: Surveillance, Epidemiology, and End Results
SREs: Skeletal-related events
