# Supplementary figures and images for: Construction and validation of web-based nomograms for detecting and prognosticating in prostate adenocarcinoma with bone metastasis
Source: Sci Rep. 2022 Nov 3;12:18623. doi: 10.1038/s41598-022-23275-w (PMC9633700; doi:10.1038/s41598-022-23275-w)

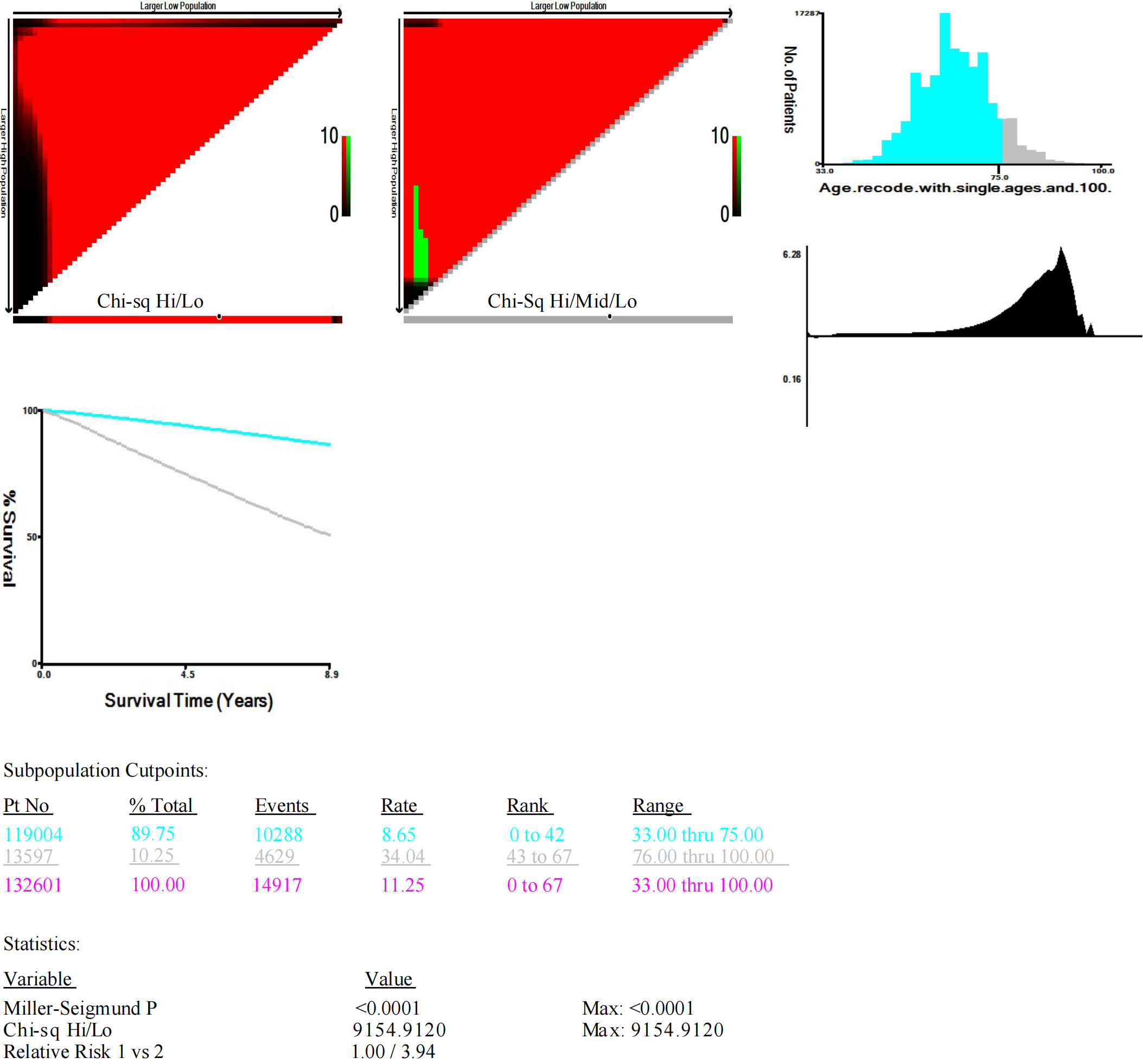

Supplement: Supplementary file 1 — Supplementary Figure S1. [file 41598_2022_23275_MOESM1_ESM.tif]

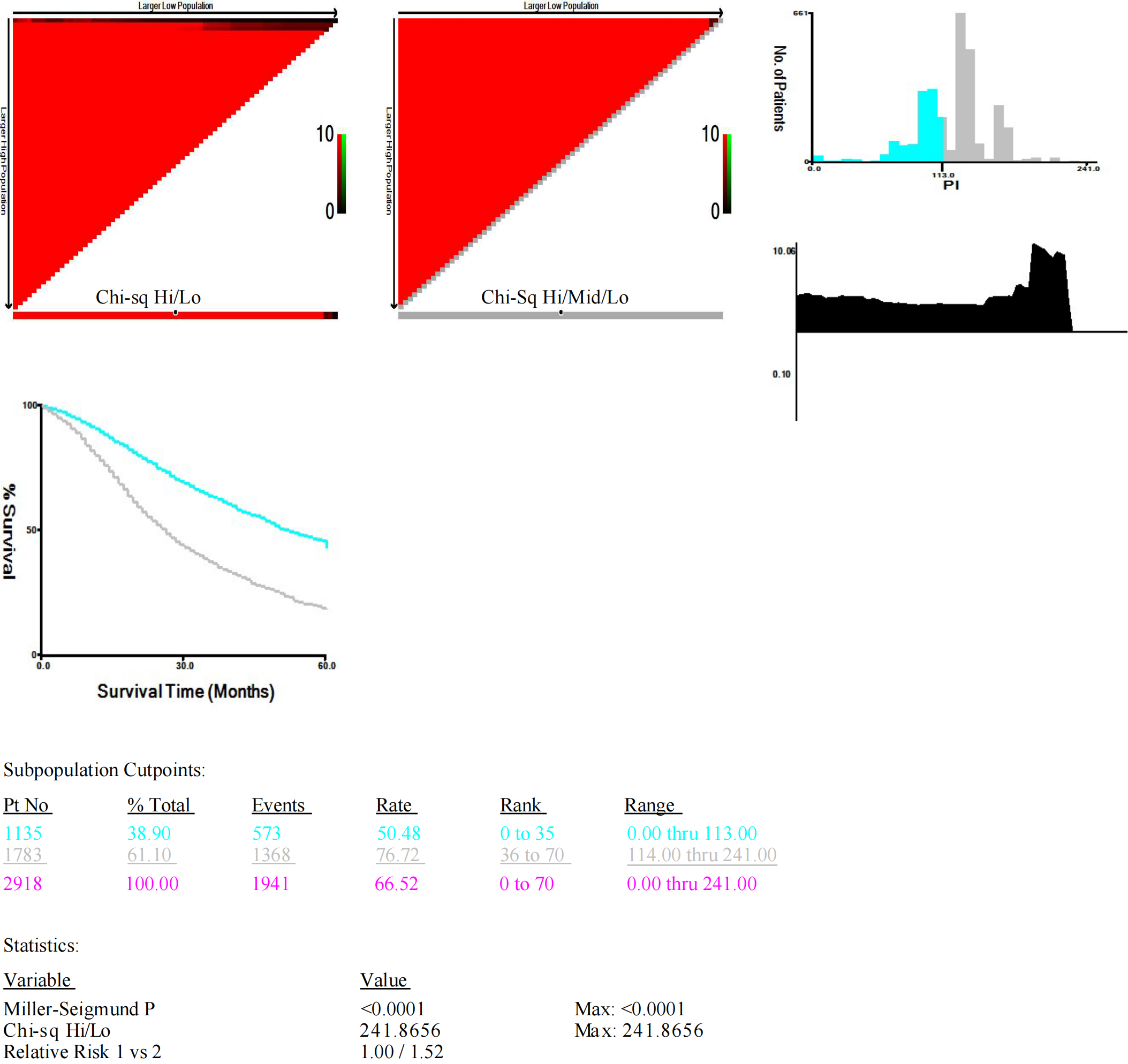

Supplement: Supplementary file 2 — Supplementary Figure S2. [file 41598_2022_23275_MOESM2_ESM.tif]

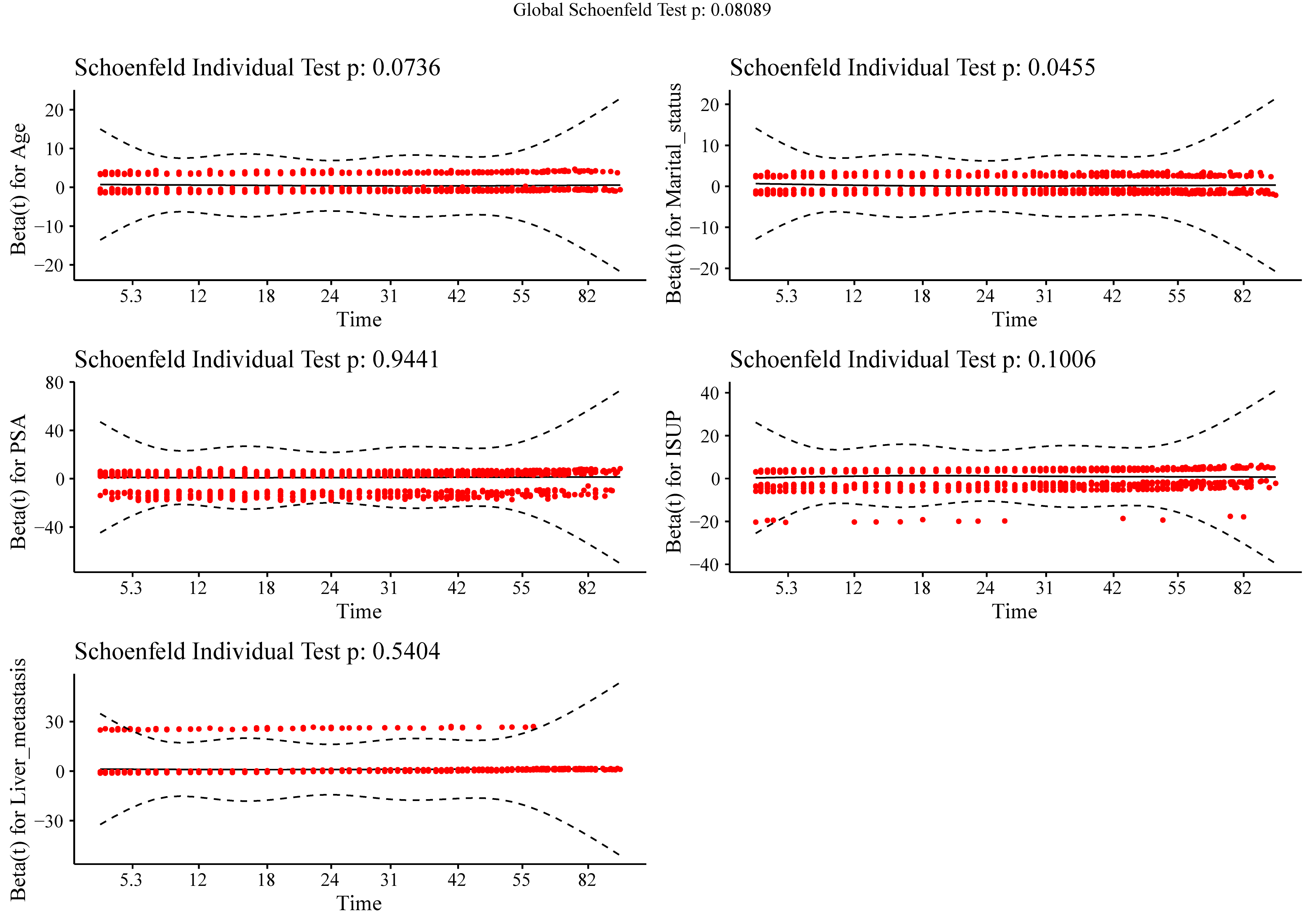

Supplement: Supplementary file 3 — Supplementary Figure S3. [file 41598_2022_23275_MOESM3_ESM.tif]
